# Early-life stature, preschool cognitive development, schooling attainment, and cognitive functioning in adulthood: a prospective study in four birth cohorts

**DOI:** 10.1016/S2214-109X(22)00448-X

**Published:** 2022-12-13

**Authors:** Aryeh D Stein, Linda S Adair, Georgina Donati, Charlotte Wray, Linda M Richter, Shane A Norris, Alan Stein, Reynaldo Martorell, Manuel Ramirez-Zea, Ana M B Menezes, Joseph Murray, Cesar Victora, Nanette Lee, Isabelita Bas, COHORTS Group, Alysse Kowalski, Ann DiGirolamo, Gaia Scerif, Feziwe Mpondo, Delia Belleza, Lukhanyo Nyati, Clive Osmond, Judith Rafaelita Borja, Delia Carba, Tita Lorna Perez, Sonny Agustin Bechavda, Maria F Kroker-Lobos, Jithin S Varghese, Fernando C Barros, Fernando P Hartwig, Bernardo L Horta, Fernando C Wehrmeister

**Affiliations:** a Hubert Department of Global Health, Rollins School of Public Health, Emory University, Atlanta, GA, USA; b Department of Nutrition, Gillings School of Global Public Health, University of North Carolina at Chapel Hill, Chapel Hill, NC, USA; c Department of Psychiatry, University of Oxford, Oxford, UK; d DSI-NRF Centre of Excellence in Human Development, University of the Witwatersrand, Johannesburg, South Africa; e SAMRC Developmental Pathways for Health Research Unit, University of the Witwatersrand, Johannesburg, South Africa; f MRC/Wits Rural Public Health and Health Transitions Research Unit (Agincourt), School of Public Health, Faculty of Health Sciences, University of the Witwatersrand, Johannesburg, South Africa; g Global Health Research Institute, School of Human Development and Health & NIHR Southampton Biomedical Research Centre, University of Southampton, Southampton, UK; h INCAP Research Center for Prevention of Chronic Diseases, Institute of Nutrition of Central America and Panama, Guatemala City, Guatemala; i Postgraduate Program in Epidemiology, Federal University of Pelotas, Pelotas, Brazil; j Human Development and Violence Research Centre, Federal University of Pelotas, Pelotas, Brazil; k USC-Office of Population Studies Foundation, University of San Carlos, Talamban, Cebu City, Cebu, Philippines

## Abstract

**Background:**

Nutrition is important for growth and brain development and therefore cognitive ability. Growth faltering in early childhood, an important indicator of early adversity, is associated with poorer developmental outcomes, some into adulthood, but this association probably reflects early-life deprivation. We aimed to investigate the associations between early-life stature, child IQ, and adult IQ.

**Methods:**

In this cohort study, we used prospective longitudinal data collected in four birth cohorts from Brazil (born in 1993), Guatemala (born in 1969–77), the Philippines (born in 1983–84), and South Africa (born in 1990). Using multivariable linear models, we estimated the relative contributions of early-life stature, child IQ, and schooling (highest school year completed) to adult IQ, including interaction effects among the early-childhood measures and schooling.

**Findings:**

We included 2614 individuals in the analysis. Early-life stature was associated with adult IQ (range across eight site-by-sex groups –0·14 to 3·17 IQ points) and schooling (–0·05 to 0·77 years) per height-for-age Z-score. These associations were attenuated when controlling for child IQ (–0·86 to 1·72 for adult IQ and –0·5 to 0·60 for schooling). The association of early-life stature with adult IQ was further attenuated when controlling for schooling (–1·86 to 1·21). Child IQ was associated with adult IQ (range 3·91 to 10·02 points) and schooling (0·25 to 1·30 years) per SD of child IQ in all groups; these associations were unattenuated by the addition of early-life stature to the models. The interaction between schooling and child IQ, but not that between schooling and early-life stature, was positively associated with adult IQ across groups.

**Interpretation:**

The observed associations of early-life stature with adult IQ and schooling varied across cohorts and sexes and explained little variance in adult IQ beyond that explained by child IQ. These findings suggest that interventions targeted at growth for health and early development are important. Our results are consistent with the inference that improving long-term cognitive outcomes might require interventions that more specifically target early cognitive ability.

**Funding:**

Bill & Melinda Gates Foundation.

## Introduction

Improving early nutrition and growth has been a major focus of global efforts to improve child outcomes. Growth faltering is widely accepted as a marker of deprivation, and the prevalence of stunting is often used as a proxy for the potential loss of human capital.^1^ Although associations between poor growth, cognitive ability, and schooling have been shown both cross-sectionally and longitudinally,^2^, ^3^, ^4^, ^5^, ^6^ effect sizes vary by age and sex, poverty levels, and the outcome measures assessed, and associations tend to attenuate over time.^5^, ^7^, ^8^, ^9^ Although previous studies have shown fairly consistent small-to-medium effect sizes,^10^ the relationship might be confounded by socioeconomic factors. Growing up in poverty is associated with both poorer growth and poorer cognitive development, which in turn are associated with lower academic attainment and earnings in adulthood.^3^, ^6^, ^10^, ^11^ When poverty is controlled for, effect sizes between growth and cognitive ability in some studies are reduced, whereas in others, using familial matched controls, the association disappears altogether.^4^ This finding could be due to familial similarities in nutritional deficits, but other familial similarities in genetic and environmental factors are also likely to influence cognitive ability. For example, in one study, parental capacity (measured by parental education and child dependency ratio) and resources explained more variance in childhood IQ than did birthweight and early height, and birthweight was a better predictor of early height than was parental capacity.^12^

Although some nutritional interventions aimed at improving growth have also improved cognitive outcomes (albeit with small effect sizes), the effects of cognitive interventions in these same studies have been up to five times larger.^9^ Furthermore, schooling has been shown to moderate the effects of poverty on cognitive development and academic achievement.^13^ A meta-analysis across studies in high-income countries showed that schooling improves IQ^14^ and that a generational effect of schooling exists on increasing population levels of IQ and intellectual capital.^15^ Collectively, these studies suggest that (1) physical growth is related to but distinct from cognitive development; (2) once basic nutritional needs are met, cognitive development might be more responsive to socio-cognitive intervention than to nutritional intervention; and (3) socio-cognitive determinants and interventions are important for ensuring effective and long-term effects on cognitive ability. In low-income and middle-income countries (LMICs), greater emphasis has been placed on interventions to improve growth rather than social determinants of cognitive development.^12^, ^16^ Although nutrition is necessary for the developing brain^17^ and stunting is an important population-level marker of healthy development, it is important to disentangle the relative specific contributions of stature, early IQ, and cognitive interventions, in this case schooling, on later cognitive ability.

**Research in context**

**Evidence before this study**

We did a review of the literature regarding growth, child development, cognition, and schooling published from 1990 to 2021. Observational cohort and intervention studies and meta-analyses consistently find associations between growth and cognitive development. However, increasing evidence exists that variation in early cognitive ability can be better explained by factors such as parental schooling than by birthweight or growth. Furthermore, meta-analyses have shown that predictors of growth faltering only partially overlap with the predictors of early cognition. Longitudinal studies have also shown an association between growth and the number of years a child remains in school. No studies that we know of have shown that early cognitive ability and growth relate differentially to years of schooling and adult cognitive ability.

**Added value of this study**

This study uses data from longitudinal cohort studies in four low-income or middle-income countries: Brazil, Guatemala, the Philippines, and South Africa. We extend the findings that determinants of early-life stature and IQ are only partially shared to show that adult IQ and schooling outcomes are better explained by early IQ than by early-life stature. Although early-life stature is associated with both outcomes in some site-by-sex comparisons, these associations are attenuated by the addition of early IQ and schooling to the models. We show that highest schooling year completed is also better predicted by child IQ than by stature and that associations between schooling and stature are substantially attenuated by the addition of child IQ. Schooling is also a good example of an intervention that has been shown to have a positive effect on cognitive development and child IQ and schooling independently and also predict adult IQ. By examining these associations separately and together across four different sites and among both sexes, we could show the robustness of cognitive predictors and schooling for adult outcomes.

**Implications of all the available evidence**

The policy implications for the collective evidence are extensive. Many studies show that physical, mental, and emotional stimulation are key to early childhood cognitive development. We showed that this early cognitive development, as well as subsequent schooling, is independently important for adult IQ. However, stature does not contribute substantial variance to adult IQ beyond its association with these two factors. This finding suggests that, although stature is an important marker for early adverse factors influencing cognitive development, policy that is designed to specifically improve cognitive development requires resources in addition to policy that targets nutrition and health*,* focused on the social and cognitive contexts that have been shown to be important. How health and nutrition can be combined with cognitive, social, and emotional care for optimum child development has been extensively elaborated within the nurturing care framework. This study provides further evidence of the long-term benefits of these joint influences.

We used prospective data collected through the Consortium of Health-Oriented Research in Transitioning Societies (COHORTS) collaboration^18^ to assess the associations between early-life stature, child IQ, schooling, and adult IQ . Supporting early child development is a key target of the Sustainable Development Goals. Thus, understanding the common and distinct long-term consequences of early life growth faltering and poor cognitive development are important to the goal of ensuring that all children reach their full developmental potential.^3^, ^11^

**Figure 1:**
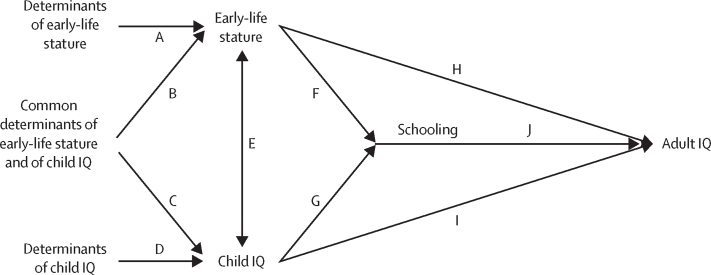
Conceptual model of the relationship between early life determinant and adult cognitive outcomes We propose that early-life stature and child IQ are correlated and have both shared and specific determinants. Associations between child stature, schooling, and adult IQ are at least partly due to this correlation. Pathways from the early-life determinants to schooling and adult IQ that do not go through early-life stature or child IQ are not shown.

We hypothesised that the association between child stature and schooling (paths F and G) and adult IQ (paths H and I) is at least partly explained by the correlation between early-life stature and child IQ (figure 1). Furthermore, we hypothesised that schooling (a cognitive intervention) will attenuate the associations between early measures and adult IQ, but with both child IQ and schooling independently predicting adult IQ (paths H, I, and J; figure 1). Finally, we hypothesised that schooling will moderate the associations of child IQ but not early-life stature with adult IQ (paths H and I, I*J, and H*J; figure 1).

## Methods

### Study design and data sources

In this prospective cohort study, we analysed both previously and newly collected survey data from four of the six birth cohorts that constitute COHORTS.^18^ Most of the data, including covariates, were derived from earlier rounds of data collection, although adult IQ data came from newly collected survey data (appendix p 4). The Brazilian cohort includes individuals born in 1993 in Pelotas.^19^ The Institute of Nutrition of Central America and Panama Nutritional Trial Cohort were all born in one of four villages in Guatemala between 1962 and 1977.^20^ The Cebu Longitudinal Health and Nutrition Survey from the Philippines includes individuals born between 1983 and 1984 in Cebu.^21^ The Birth to Thirty cohort were all born in Soweto or Johannesburg, South Africa, in 1990.^22^ At cohort inception, South Africa and Brazil were classified as middle-income countries, and Guatemala and the Philippines were low-income countries. The Pelotas and Soweto cohorts were exclusively urban, the Cebu cohort was a mix of urban and rural participants, and the Guatemala cohort was rural. The New Delhi Birth Cohort and the 1982 Pelotas birth cohort, which are part of the COHORTS collaboration, were not included in this analysis because of the absence of measures of early-life cognition. All fieldwork followed procedures approved by local ethics review committees and all participants or parents, as appropriate, provided written informed consent. The present analyses were approved by the Emory University Institutional Review Board (95960).

### Data collected in childhood

#### Child IQ

IQ remains a widely accepted and reliable index of individual differences in general cognitive ability and marker of human-capital outcomes. In Brazil, a short form of the Wechsler Preschool Intelligence Scale (WPPSI),^24^ consisting of two verbal subtests (comprehension and arithmetic) and two non-verbal subtests (figure completion and construction with cubes), adapted and translated into Portuguese, was administered at a mean age of 4·5 years. In Guatemala, a comprehensive battery of 10–22 modules taken from various sources including the WPPSI was administered annually from age 3 to 7 years.^25^ In the Philippines, the Philippine Nonverbal Intelligence Test (PNIT)^26^ was administered at a mean age of 8·5 years. Modelled on the Raven's Colored Progressive Matrices (RCPM),^27^ the PNIT consists of 100 items requiring the child to indicate which target object is different from others, progressing from concrete to more abstract tasks. In South Africa, the RCPM was administered at a mean age of 7·8 years.

Within each site, we converted the test results into a standardised distribution mean of 100 (SD 15). For Guatemala, we first standardised the test results obtained within each year of age and used the standardised values for the administration closest to age 7 years.^12^ These harmonised scores permit analysis within each site but do not reference any external norm.

#### Early-life stature

We obtained measurements in childhood using site-specific protocols as described elsewhere.^20^, ^21^, ^22^, ^28^ For this analysis we used measures of stature that were obtained at age 1 year for Brazil and age 2 years for Guatemala, the Philippines, and South Africa. For convenience, we refer to this measure as early-life stature. Stature was expressed as height-for-age Z scores (HAZ) using the WHO Growth Standards.^29^ We also created a category measure that divided the HAZ groups (<–3, ≥–3 to <–2, ≥–2 to –1, and ≥–1). No individuals from Guatemala had a HAZ of 1 or greater and no individuals from Brazil had a HAZ below –3.

### Data collected in adulthood

Adult data collection^28^, ^30^ was done in 2011 in Brazil (mean age 18·5 years) and in 2018–19 in the other three cohorts (Mean age 45 years in Guatemala, 35 years in the Philippines, and 28 years in South Africa). For the Brazil cohort, a large proportion of participants were still in tertiary education; the other cohorts had largely completed their schooling before collection of the adult data.

#### Schooling

Schooling, as defined by highest formal schooling year completed, was assessed by interview and was used as a continuous measure for the main analyses. Highest school year attained was also categorised as a binary variable, representing the most common thresholds in each site: low (did not compete primary [Guatemala] or secondary school [Brazil, Philippines, and South Africa]) versus high (completed primary [Guatemala] or secondary school [Brazil, Philippines, and South Africa]).

#### Adult IQ

In Brazil, the arithmetic, digit symbol, similarities, and picture completion subtests of the Wechsler Adult Scale of Intelligence test (3rd version) were administered.^28^ In Guatemala, the Philippines, and South Africa, the Raven's Standard Progressive Matrices test was administered.^27^ In Guatemala, only sections A–C of the test were used because of a previously shown inability to proceed beyond section C,^42^ for a maximum score of 36 points. In the Philippines and South Africa, sections A–E were used, for a maximum score of 60 points. We standardised the distribution within each cohort and by sex to a mean of 100 and an SD of 15 to remove between-cohort differences that might relate to language of administration, context, or tests administered. These harmonised scores permit analysis within each site but do not reference any external norm.

### Covariates

We included previously established determinants of early-life statures and child IQ as covariates.^12^, ^31^ These determinants included maternal height (cm), maternal schooling, birth order (first, second, third, or fourth or later), household socioeconomic status at the time of the cohort participant's birth (represented as quintiles of the site-specific distribution of wealth, computed within each site as the first component of an asset-based principal component index calculated from a list of assets available in the household), and (for Guatemala) birth year and intervention group were provided by each cohort research team.^20^

### Statistical analysis

We restricted analyses to participants with complete information for early-life stature, child IQ, schooling, and adult IQ. For descriptive analyses, we calculated means and SDs for continuous variables and proportions for categorical variables.

We built a series of models using ordinary least squares linear regression to estimate the strength of the associations between the factors in our conceptual model. First, we estimated the separate associations of early-life stature and child IQ with highest formal schooling year completed (F and G in figure 1) and with adult IQ (H and I). Next, to assess mutual confounding, we assessed the extent to which the estimates for early-life stature and child IQ on the associations with adult IQ were attenuated when the other factor was entered into the model (EF and EG). We then estimated the association between schooling and adult cognition (J). To estimate the extent to which schooling attenuated the association between early-life stature, child IQ, and adult IQ, we estimated the associations between early-life stature, child IQ, and adult IQ, controlling for schooling (FJ, GJ, EFJ, and EGJ). Finally, to examine whether the associations between early-life stature and adult IQ and between child IQ and adult IQ were differentially modified by schooling, we considered interaction terms between early-life stature and schooling and between child IQ and schooling. Finally, we compared adult IQ scores across categories of child stature, child IQ, and schooling. All models were adjusted for a consistent set of early-life characteristics, including maternal schooling, height (cm), wealth (quintile [categorical]), and birth order (first, second, third, or fourth or later). Additionally, analyses for the Guatemala data were adjusted for treatment assignment in the original intervention study and for birth year given the wide age range. We considered a p value of less than 0·05 to indicate statistical significance and did not make any adjustments for multiple comparisons. We present point estimates and 95% CIs. Interaction terms were considered statistically significant at a p value of less than 0·05. We used Stata (version 15) for all analyses.

### Role of the funding source

The funder of the study had no role in study design, data collection, data analysis, data interpretation, or writing of the report.

## Results

We included 2614 individuals in the analysis (table 1). Levels of maternal and own schooling were lowest in Guatemala and highest in South Africa (table 1). Early-life stature was lowest in Guatemala and the Philippines (table 1). Correlations between early-life stature and child IQ were positive in all cohorts (in Brazil *r*=0·286 for males and *r*=0·232 females, in Guatemala *r*=0·247 for males and *r*=0·189 for females, in the Philippines *r*=0·249 for males and *r*=0·329 for females, and in South Africa *r*=0·102 for males and *r*=0·055 for females).

**Table 1:** Selected characteristics of the study sample by site and sex

		**Brazil**		**Guatemala**		**The Philippines**		**South Africa**	
		Female (n=249)	Male (n=201)	Female (n=229)	Male (n=196)	Female (n=590)	Male (n=686)	Female (n=247)	Male (n=216)
Maternal schooling, years		7·2 (3·9)	7·0 (3·9)	1·2 (1·5)	1·4 (1·5)	6·6 (3·0)	6·9 (3·3)	9·7 (2·5)	9·8 (2·3)
Maternal height, cm		160 (7·6)	160 (7·6)	148 (5·4)	148 (4·8)	150 (5·0)	150 (4·9)	158 (5·6)	158 (6·1)
Birth order									
	First	60 (29·9%)	85 (34·1%)	37 (18·9%)	35 (15·3%)	152 (22·4%)	128 (21·7%)	83 (38·3%)	96 (38·9%)
	Second	68 (33·8%)	70 (28·1%)	20 (10·2%)	30 (13·1%)	135 (19·7%)	135 (22·9%)	58 (26·9%)	72 (29·2%)
	Third	32 (15·9%)	52 (20·9%)	24 (12·2%)	31 (13·5%)	137 (20·0%)	110 (18·6%)	43 (19·9%)	42 (17·0%)
	Fourth or later	41 (20·4%)	42 (16·9%)	115 (58·7%)	133 (58·1%)	262 (38·2%)	217 (36·8%)	32 (14·8%)	37 (15·0%)
Early-life stature, HAZ		−0·17 (1·26)	−0·24 (1·48)	−2·94 (1·12)	−3·02 (1·16)	−2·42 (1·13)	−2·47 (1·12)	−1·12 (0·99)	−1·37 (1·04)
Child IQ		102·7 (15·9)	99·7 (15·6)	102·4 (12·5)	104·0 (14·4)	100·0 (14·6)	98·3 (14·8)	98·5 (12·6)	99·9 (15·2)
Age at adult assessment, years		18	18	47·4 (2·09)	47·4 (1·90)	34·4 (0·51)	34·4 (0·52)	28·5 (0·37)	28·4 (0·35)
Schooling, years		10·4 (2·2)	9·3 (2·6)	4·8 (3·9)	5·8 (3·8)	10·7 (2·6)	9·8 (3·0)	12·0 (1·2)	11·6 (1·4)
Adult IQ		101·9 (14·7)	100·9 (16·3)	97·9 (14·3)	104·8 (15·1)	99·7 (15·2)	100·2 (14·8)	99·4 (15·1)	102·8 (13·0)

Data are mean (SD) or n (%). Early-life stature was measured at age 2 years in all cohorts except in Pelotas (1 year). Child IQ was measured at age 3–7 years (Guatemala), 4 years (Brazil and South Africa), and 8·5 years (the Philippines). Age at assessment in Brazil recorded in integer years and all were 18 years old. HAZ=height-for-age Z score.

**Figure 2:**
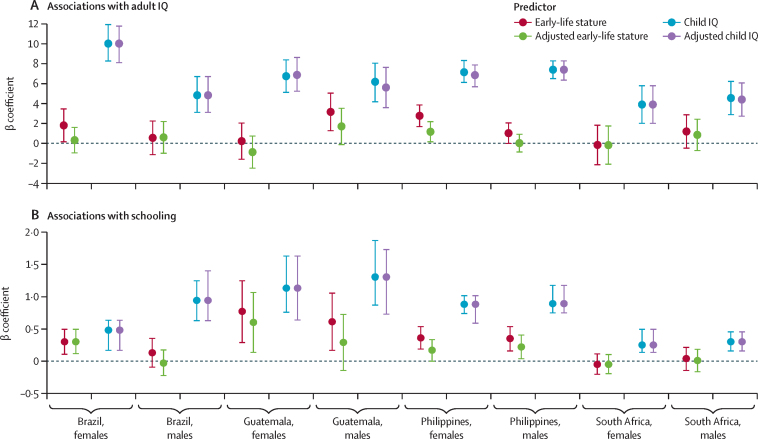
Associations between early-life stature and child IQ with adult IQ and schooling Plotted on the y axis are the unadjusted and adjusted β coefficients with their 95% confidence intervals for each regression model. Unadjusted models show the β coefficients for a regression model including either child IQ or early-life stature and the covariates. Adjusted models include both early-life stature and child IQ and therefore adjustment refers to the inclusion of the other main variable of interest in the regression model. All data including p values are shown in the appendix (p 2).

Early-life stature was significantly positively associated with adult IQ in four of eight site-by-sex comparisons (figure 2A; appendix p 2), ranging across all eight comparisons from –0·14 to 3·17 IQ points per height-for-age Z-score and being strongest among males in Guatemala and females in the Philippines (figure 2A; appendix p 2). Child IQ was significantly positively associated with adult IQ in all eight site-by-sex comparisons (figure 2A; appendix p 2), ranging from 3·91 to 10·02 points per SD of child IQ. In mutually adjusted models that examined the joint association between early-life stature and child IQ, child IQ was significantly positively associated with adult IQ in all eight site-by-sex groups, whereas early-life stature remained significantly associated with adult IQ in only the Philippines for females (figure 2A; appendix p 2). Early-life stature was significantly positively associated with schooling in Guatemala (males and females), the Philippines (males and females), and Brazil (females), but not in South Africa (figure 2B; appendix p 2; range 0·30–0·77 years per height-for-age Z-score). Child IQ was positively associated with schooling in all eight site-by-sex groups (figure 2B; appendix p 2), ranging from 0·25 to 1·30 years per SD of child IQ. In mutually adjusted models, the coefficients for early-life stature were significantly attenuated (although not always to 0) for three site-by-sex groups. Those for child IQ, on the other hand, were unchanged.

Schooling was positively associated with adult IQ, with the difference in adult IQ between those completing and not completing the schooling threshold ranging from 9·3 points (males in South Africa) to 16·3 points (males in Brazil; appendix p 4). The association was significant in models including both early-life stature and child IQ (table 2). The association between early-life-stature and adult IQ was attenuated by adjustment for schooling but was significant in three site-by-sex comparisons (table 2). The association between child IQ and adult IQ was also attenuated by adjustment for schooling but was statistically significant in all site-by-sex groups (table 2).

**Table 2:** Regression analyses assessing the extent to which schooling attenuates and moderates the associations of early-life stature and child IQ with adult IQ

			**Brazil**		**Guatemala**		**The Philippines**		**South Africa**	
			Female (n=249)	Male (n=201)	Female (n=229)	Male (n=196)	Female (n=590)	Male (n=686)	Female (n=247)	Male (n=216)
**Hypothesis: controlling for schooling will attenuate the strength of the association between early-life stature and adult cognition but not childhood cognition and adult cognition**										
Adult IQ										
	Path J									
		Schooling	2·86 (2·08 to 3·63)	3·75 (3·03 to 4·47)	0·74 (0·58 to 0·89)	0·83 (0·64 to 1·03)	2·40 (2·08 to 2·72)	1·85 (1·60 to 2·11)	2·75 (1·79 to 3·71)	1·69 (0·89 to 2·48)
	Paths H and J									
		Early-life stature	1·35 (0·02 to 2·68)	−0·29 (−1·90 to 1·31)	−1·35 (−2·90 to 0·21)	1·88 (0·22 to 3·53)	1·67 (0·72 to 2·62)	0·19 (−0·72 to 1·10)	0·05 (−1·82 to 1·91)	1·12 (−0·49 to 2·72)
		Schooling	3·70 (2·99 to 4·42)	2·89 (2·08 to 3·70)	2·06 (1·64 to 2·48)	2·12 (1·59 to 2·65)	3·06 (2·63 to 3·49); p<0	2·45 (2·11 to 2·79)	4·16 (2·72 to 5·66)	2·55 (1·34 to 3·76)
	Paths I and J									
		Child IQ	7·31 (5·57 to 9·22)	3·90 (2·18 to 5·62)	4·88 (3·38 to 1·25)	3·74 (1·87 to 5·62)	4·96 (3·94 to 5·99)	5·77 (4·74 to 6·66)	2·77 (0·88 to 4·54)	3·95 (2·28 to 5·62)
		Schooling	2·63 (1·92 to 3·33)	2·48 (1·66 to 3·30)	1·59 (1·19 to 1·99)	1·82 (1·27 to 2·37)	2·46 (2·03 to 2·89)	1·77 (1·45 to 2·10)	3·63 (2·14 to 5·13)	1·99 (0·81 to 3·17)
	Paths H, J, and I									
		Early-life stature	0·41 (−0·71 to 1·54)	−0·14 (−1·67 to 1·38)	−1·86 (−3·29 to −0·44)	1·21 (−0·45 to 2·86)	0·78 (−0·14 to 1·69)	−0·35 (−1·18 to 0·47)	0·01 (−1·82 to 1·85)	0·84 (−0·69 to 2·38)
		Child IQ	7·31 (5·41 to 9·06)	3·90 (2·18 to 5·62)	5·13 (3·63 to 6·63)	3·46 (1·44 to 5·33)	4·82 (3·65 to 5·84)	5·77 (4·88 to 6·66)	2·77 (0·88 to 4·54)	3·80 (2·13 to 5·47)
		Schooling	2·63 (1·93 to 3·34)	2·50 (1·66 to 3·34)	1·68 (1·28 to 2·08)	1·78 (1·23 to 2·33)	2·43 (2·01 to 2·86)	1·78 (1·46 to 2·11)	3·63 (2·14 to 5·13)	1·98 (0·80 to 3·16)
**Hypothesis: the associations of early-life stature and of child IQ with adult IQ will be moderated by schooling**										
Adult IQ										
	Paths H, J, and H*J									
		Early-life stature	1·96 (−1·93 to 5·85)	4·38 (−1·48 to 10·23)	−0·56 (−2·70 to 1·58)	0·88 (−2·08 to 3·84)	−0·15 (−3·70 to 3·40)	0·47 (−2·32 to 3·26)	3·24 (−20·23 to 26·71)	3·61 (−11·52 to 18·75)
		Schooling	3·68 (2·98 to 4·37)	2·53 (1·56 to 3·50)	1·47 (0·27 to 2·66)	2·63 (1·26 to 4·01)	3·50 (2·57 to 4·43)	2·38 (1·60 to 3·15)	3·89 (1·27 to 6·52)	2·14 (−0·58 to 4·87)
		Early-life stature*schooling (interaction)	−0·06 (−0·49 to 0·36)	−0·44 (−1·06 to 0·17)	−0·22 (−0·63 to 0·19)	0·19 (−0·28 to 0·65)	0·18 (−0·15 to 0·51)	−0·03 (−0·29 to 0·24)	−0·27 (−2·24 to 1·70)	−0·22 (−1·52 to 1·09)
	Paths I, J, and I*J									
		Child IQ	2·70 (−3·02 to 8·43)	−2·96 (−11·08 to 5·15)	2·25 (0·00 to 4·50)	1·02 (−2·30 to 4·32)	0·00 (−3·50 to 3·36)	5·33 (2·81 to 7·99)	7·18 (−6·30 to 20·66)	14·29 (0·30 to 28·42)
		Schooling	−0·25 (−4·02 to 3·52)	−1·42 (−6·41 to 3·57)	−3·44 (−6·56 to −0·32)	−1·65 (−5·11 to 1·81)	−0·66 (−2·75 to 1·42)	1·54 (−0·06 to 3·15)	6·55 (−2·39 to 15·50)	7·86 (−0·07 to 15·78)
		Child IQ*schooling (interaction)	0·48 (−0·16 to 1·11)	0·62 (−0·16 to 1·40)	0·63 (0·25 to 1·00)	0·43 (0·00 to 0·86)	0·44 (0·15 to 0·73)	0·00 (−0·15 to 0·30)	−0·38 (−1·39 to 0·76)	−0·91 (−2·03 to 0·30)
	Paths H, I, I*J, and H*J									
		Early-life stature	1·46 (−2·03 to 4·96)	7·02 (1·01 to 13·03); p<0·05	−1·5 (−3·50 to 0·49)	1·0 (−2·06 to 4·06)	0·47 (−3·08 to 4·02)	−0·02 (−2·70 to 2·67)	3·31 (−19·87 to 26·48)	1·11 (−13·47 to 15·69)
		Schooling	−0·81 (−4·69 to 3·07)	−2·16 (−7·01 to 2·69)	−2·79 (−6·16 to 0·57)	−1·54 (−5·88 to 2·79)	−0·53 (−3·17 to 2·11)	1·38 (−0·67 to 3·43)	6·32 (−2·80 to 15·45)	7·38 (−1·29 to 16·05)
		Child IQ	1·75 (−4·13 to 7·47)	−2·96 (−10·61 to 4·52)	2·75 (0·50 to 5·00)	0·60 (−3·02 to 4·38)	−0·15 (−3·80 to 3·65)	5·33 (2·52 to 7·99)	7·31 (−6·30 to 20·92)	13·53 (−0·76 to 27·82)
		Early-life stature*schooling (interaction)	−0·09 (−0·47 to 0·28)	−0·68 (−1·31 to −0·06)	0·00 (−0·38 to 0·38)	0·06 (−0·42 to 0·54)	0·03 (−0·30 to 0·36)	−0·03 (−0·29 to 0·22)	−0·28 (−2·22 to 1·67)	−0·03 (−1·29 to 1·23)
		Child IQ*schooling (interaction)	0·64 (−0·00 to 0·64)	0·62 (−0·16 to 1·40)	0·50 (0·13 to 0·88)	0·45 (−0·00 to 1·06)	0·44 (0·15 to 0·73)	0·00 (−0·15 to 0·30)	−0·38 (−1·51 to 0·76)	−0·76 (−1·98 to 0·30)

Data are β (95% CI). Paths are detailed in figure 1. Early-life stature was measured at age 2 years except in Pelotas (at age 1 year). Child IQ was measured at age 3–7 years (Guatemala), 4 years (Brazil and South Africa), and 8·5 years (the Philippines). Estimates are years of schooling per height-for-age Z score or per site-specific and sex-specific SD of child IQ. Analyses were adjusted for maternal schooling attainment (years), height (cm), wealth (quintile, categorical), and birth order (first, second, third, or fourth or later). Results for Guatemala were also adjusted for birth year and treatment assignment. Results for Brazil were weighted to reflect sampling.

**Figure 3:**
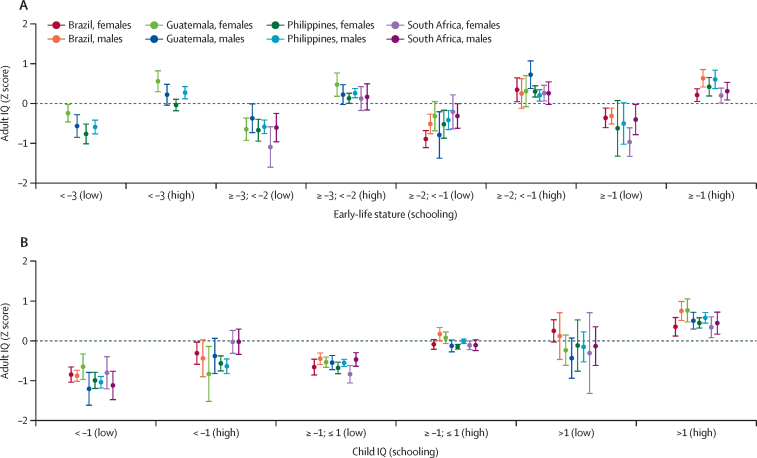
Adult IQ according to categories of early-life stature (A) and child IQ (B) and schooling in four birth cohorts, by sex Bars indicate mean adult IQ by site and sex, within categories of HAZ at around age 2 years and schooling attainment. HAZ is categorised as less than –3, –3 to less than –2, –2 to –1, –1 or greater. No individuals from Guatemala had a HAZ of 1 or greater and no individuals from Brazil had a HAZ below –3. Child IQ was measured at age 4·5 years (Brazil), 4–7 years (Guatemala), 8·5 years (the Philippines), and 4·5 years (South Africa). Child IQ was categorized as less than –1, –1 to +1, or greater than +1 SD of the site-specific distribution. Schooling was categorised as low (did not compete primary [Guatemala] or secondary school [Brazil, the Philippines, and South Africa]) versus high (completed primary [Guatemala] or secondary school [Brazil, the Philippines, and South Africa]). HAZ=height-for-age Z score.

Interaction terms for early-life stature and schooling were not statistically significant, and those for child IQ and schooling were statistically significant for three of the eight groups (table 2). Individuals with higher schooling had higher IQ than those with lower schooling did, but child HAZ was not strongly associated with adult IQ (figure 3A). However, child IQ and schooling attainment were independently associated with adult IQ, with consistent associations across site-by-sex comparisons (figure 3B).

## Discussion

This study showed that both schooling and adult IQ were more strongly associated with child IQ than they were with early-life stature. Further, the associations between early-life stature and later outcomes were almost entirely attenuated by controlling for child IQ and schooling. Because previous work has shown that childhood cognitive ability is more strongly influenced by social factors such as parental capacity and resources than by early childhood growth,^12^ and that improving early nutrition alone does not necessarily improve cognition,^9^ this study provides further evidence that a global focus on growth and nutrition, although crucial for health and useful as a marker for deprivation, requires supplementation with development-specific interventions to support cognitive development.

Previous studies from these and other cohorts have shown that larger birthweight and greater weight gain in the first 2 years of life predict staying in school.^2^, ^10^, ^32^ These studies show the utility of birthweight and early growth as a general indicator of child circumstances. In this study, the predictive power of early-life stature on schooling was attenuated by adjustment for child IQ, while child IQ consistently predicted how long individuals stayed in school in all cohorts for both sexes, even when controlling for early-life stature. This finding supports previous work showing that early cognitive development sets a foundation for the benefits of later schooling^3^ and the weak correlation between early-life stature and child IQ supports previous literature suggesting some common determinants.^12^, ^31^

Many observational studies have shown an association between growth faltering and poorer adult cognitive outcomes.^4^, ^6^ However, the results of intervention trials, which are more robust against confounding, have proven inconclusive. When interventions have improved both weight and cognitive outcomes concurrently, these improvements did not necessarily persist.^8^, ^33^, ^34^ When improvements did endure, the effect was often ascribed to either the combination of nutritional intervention with parenting interventions to encourage cognitive stimulation^35^ or cognitive stimulation alone.^36^, ^37^ Our study found that where an association between early-life stature and adult IQ existed, this association was attenuated by child IQ and schooling. This finding suggests that although early-life stature remains an important marker for adult cognitive outcomes, early cognitive development, along with cognitive interventions such as schooling, are better able to explain variation in adult IQ. Other research has already shown that early IQ is more strongly associated with parental capacity than with birthweight and growth^12^ and that interventions supporting nurturing care (a combination of health and nutrition, responsive care, and cognitively enriching environments across development^38^) tend to be successful and have a greater effect than do those supporting only one aspect of development.^9^

The attenuation in the association between child IQ and adult IQ when including schooling replicates previous findings.^13^, ^14^, ^39^ We found a significant positive interaction effect between child IQ and schooling for three of the eight site-by-sex groups, where schooling increased adult IQ more for those with higher child IQ.

This study used observational data and therefore was not able to make causal inferences; in particular, we cannot be sure of the direction of effect between cognitive ability and schooling. Randomised studies of this question are impractical given the long time horizon, and hence conclusions must be drawn from prospective observational data such as these. Compared with the inception cohorts, sample sizes in this study were reduced because of attrition over the decades of follow-up and the need to have complete data on the key variables for analysis, which were obtained over the life course. Schooling is a crude measure of educational inputs; information was limited to highest schooling year completed and we did not have information on the quality of the schools attended. The measures of stature and child IQ were obtained at different ages in the different cohorts and the measurement of early-life stature at younger ages than the measurement of child IQ allows for the possibility that stature causally influences child IQ. However, the weak correlations between these measures and results of previous studies showing the differential influences on each factor^9^, ^12^ suggest that this causality would fully account for our findings. Intelligence is a complex construct that is only partially measured by the tests we implemented. Although adult IQ measures were obtained at different ages in different cohorts, the stability of adult IQ in terms of individual differences (before age 60 years) suggests that we would not expect any related considerable differences in the analyses across cohorts. Child IQ measures varied between sites; the measures from Brazil and Guatemala encompassed both verbal and non-verbal IQ whereas those in South Africa and the Philippines captured only non-verbal IQ. Participants with verbal IQ measures only might be expected to show greater associations between IQ and years in schooling since other research has shown a greater association between verbal IQ and academic attainment.^40^ However, we did not find this to be the case and so these differences in IQ measures probably only introduce a small amount of additional variability compared with differences introduced by other site-specific factors. Considering the added variability introduced by different ages at which IQ was measured, as well as the tests themselves, the associations we observed are remarkably robust across sites, which suggests that these findings might have broad generalisability across LMICs.

Our analyses also have major strengths. This study used prospective data from four well-described cohorts representing various social and economic contexts. We also controlled for key confounding factors associated with children's early environment.

In conclusion, child IQ was found to be a robust predictor of later schooling and adult IQ in all models and in all site-by-sex groups. Although we did find associations between early-life stature and cognitive and schooling outcomes, the strength of the associations varied across cohorts and sexes and, importantly, early-life stature explained little variance in adult IQ beyond that explained by early IQ and schooling. These results are consistent with the inference that factors in addition to those represented by early-life stature are important for cognitive development, and that these in turn are associated with staying in school and higher levels of adult IQ. To the extent that these results represent a causal pathway, the implications of these findings on policy, aid, and intervention research are broad. If we are serious about improving long-term cognitive outcomes, we need to start investing in factors that influence early cognitive development directly, in addition to ongoing efforts to promote child nutrition and health. Many studies show that physical, mental, and emotional stimulation are key to promoting cognitive development.^38^ Together with these studies, our work provides support for a more holistic approach to promoting human capital outcomes such as those promoted by the nurturing care framework, in which health and nutrition are supplemented with opportunities for learning, protection, and responsive caregiving to provide the physical, mental, and emotional stimulation needed for healthy development.^41^

## Data sharing

Data from the Philippines are available online (https://cebu.cpc.unc.edu/datasets/). Data from the other cohorts can be requested from the investigators.

## Declaration of interests

We declare no competing interests.
