# Supplementary material for: Early-life stature, preschool cognitive development, schooling attainment, and cognitive functioning in adulthood: a prospective study in four birth cohorts
Source: Lancet Glob Health. 2022 Dec 13;11(1):e95–e104. doi: 10.1016/S2214-109X(22)00448-X (PMC9764453; doi:10.1016/S2214-109X(22)00448-X)
Supplement: Supplementary appendix [file mmc1.pdf]

THE LANCET
Global Health

Supplementary appendix

This appendix formed part of the original submission and has been peer reviewed. We post it as supplied by the authors.

Supplement to: Stein AD, Adair LS, Donati G, et al. Early-life stature, preschool cognitive development, schooling attainment, and cognitive functioning in adulthood: a prospective study in four birth cohorts. *Lancet Glob Health* 2023; **11:** e95–104.

Early life stature, preschool cognitive development, schooling attainment, and cognitive functioning in adulthood: prospective study in four birth cohorts

Professor Aryeh D Stein, PhD[1‡], Professor Linda S Adair, PhD[2], Georgina Donati, PhD[3], Charlotte Wray, PhD[3], Professor Linda M Richter, PhD[4], Professor Shane A Norris, PhD[5,6], Professor Alan Stein FRCPsych [3,7], Professor Reynaldo Martorell, PhD[1], Manuel Ramirez-Zea, PhD[8], Professor Ana M B Menezes, PhD[9], Joseph Murray, PhD[9,10], Professor Cesar Victora, MD[9], Nanette Lee, PhD[11], Isabelita Bas, MA[11] and COHORTS Group*

[1] Hubert Department of Global Health, Rollins School of Public Health, Emory University, Atlanta GA, USA
[2] Department of Nutrition, Gillings School of Global Public Health, University of North Carolina at Chapel Hill, Chapel Hill, NC, USA
[3] Department of Psychiatry, University of Oxford, Oxford, United Kingdom
[4] DSI-NRF Centre of Excellence in Human Development, University of the Witwatersrand, Johannesburg, South Africa
[5] SAMRC Developmental Pathways for Health Research Unit, University of the Witwatersrand, Johannesburg, South Africa
[6] Global Health Research Institute, School of Human Development and Health & NIHR Southampton Biomedical Research Centre, University of Southampton, United Kingdom
[7] MRC/Wits Rural Public Health and Health Transitions Research Unit (Agincourt), School of Public Health, Faculty of Health Sciences, University of the Witwatersrand, Johannesburg, South Africa
[8] INCAP Research Center for Prevention of Chronic Diseases, Institute of Nutrition of Central America and Panama, Guatemala City, Guatemala
[9] Postgraduate Program in Epidemiology, Federal University of Pelotas, Pelotas, Brazil
[10] Human Development and Violence Research Centre (DOVE), Federal University of Pelotas, Pelotas, Brazil
[11] USC-Office of Population Studies Foundation, Inc, University of San Carlos – TC, Talamban, Cebu City, Cebu, Philippines

[‡]**Corresponding Author**
Aryeh D Stein
Rollins School of Public Health
Emory University
Atlanta, GA, USA
aryeh.stein@emory.edu

Supplementary Table 1. Regression analyses assessing the extent to which associations of early-life stature with child IQ, schooling and adult IQ are attenuated by each other

Outcome		Brazil females	Brazil males	Guatemala females	Guatemala males	Philippines females	Philippines males	South Africa females	South Africa males
	N	249	201	229	196	590	686	247	216
		B (95% CI)	B (95% CI)	B (95% CI)	B (95% CI)	B (95% CI)	B (95% CI)	B (95% CI)	B (95% CI)
**Hypothesis 1: Both early-life stature and child IQ will predict schooling, and statistical adjustment for child IQ will attenuate the strength of the estimate for early-life stature, but not vice versa**									
Schooling									
	**Path F**								
	Early-life stature	0·30** (0·10,0·50)	0·13 (-0·10,0·36)	0·77** (0·28,1·25)	0·61** (0·16,1·06)	0·36*** (0·18,0·54)	0·35*** (0·15,0·54)	-0·05 (-0·21,0·12)	0·04 (-0·15,0·22)
	**Path G**								
	Child IQ	0·48** (0·16,0·64)	0·94***(0·62,1·25)	1·13*** (0·75,1·63)	1·30*** (0·86,1·87)	0·88*** (0·73,1·02)	0·89*** (0·74,1·18)	0·25*** (0·13,0·50)	0·30** (0·15,0·46)
	**Path F & G**								
	Early-life stature	0·30** (0·11,0·50)	-0·03 (-0·23,0·18)	0·60* (0·13,1·07)	0·29 (-0·15,0·73)	0·17 (-0·01,0·34)	0·22* (0·03,0·41)	-0·05 (-0·20,0·11)	0·01 (-0·17,0·19)
	Child IQ	0·48** (0·16,0·64)	0·94*** (0·62,1·40)	1·13*** (0·63,1·63)	1·30*** (0·72,1·73)	0·88*** (0·58,1·02)	0·89*** (0·74,1·18)	0·25*** (0·13,0·50)	0·30** (0·15,0·46)
**Hypothesis 2: Both early-life stature and child IQ will predict adult IQ and statistical adjustment for child IQ will attenuate the strength of the estimate for early-life stature, but not vice versa**									
Adult IQ									
	**Path H**								
	Early-life stature	1·82* (0·17,3·47)	0·57 (-1·11,2·26)	0·24 (-1·58,2·05)	3·17** (1·29,5·05)	2·78*** (1·70,3·87)	1·04* (0·01,2·07)	-0·14 (-2·12,1·84)	1·21 (-0·46,2·88)
	**Path I**								
	Child IQ	10·02***(8·27,11·93)	4·84*** (3·12,6·71)	6·75*** (5·13,8·38)	6·19*** (4·18,8·06)	7·15*** (6·13,8·32)	7·40*** (6·51,8·29)	3·91*** (2·02,5·80)	4·56*** (2·89,6·23)
	**Path H & I**								
	Early-life stature	0·34 (-0·93,1·62)	0·62 (-0·97,2·21)	-0·86 (-2·47,0·75)	1·72 (-0·10,3·54)	1·18* (0·17,2·19)	0·04 (-0·85,0·93)	-0·16 (-2·07,1·76)	0·87 (-0·71,2·44)
	Child IQ	10·02*** (8·11,11·77)	4·84*** (3·12,6·71)	6·88*** (5·25,8·63)	5·62*** (3·60,7·63)	6·86*** (5·69,7·88)	7·40*** (6·36,8·29)	3·91*** (2·02,5·80)	4·41*** (2·74,6·08)

Early-life stature measured at age 2 yr except in Pelotas (1 y)· Child IQ measured at age 3-7 yr (Guatemala); 4 yr (Bto20+, Pelotas); 8·5 yr (Cebu).

Estimates are years of schooling per HAZ or per site- and sex-specific SD of child IQ, respectively

Adjusted for maternal schooling attainment (y), height (cm), and wealth (quintile, categorical), child birth order (1, 2, 3, 4+). Results for Guatemala also adjusted for birth year and treatment assignment (atole or fresco). Results for Brazil weighted to reflect sampling.

* p<0·05; ** p<0·01; *** p<0·001

Supplementary Table 2: Flow chart of sample availability for analysis

	Brazil	Guatemala	Philippines	South Africa
Inception Cohort	5265	2392	3280	3273
IQ data collection in adulthood	4057	1268	1327	1402
Missing required early-life data	3607[2]	843[3]	51	939
Included in current analysis	450	425	1276	463

Notes

1. Reasons for loss to follow-up include death, outmigration from the study area, loss to follow-up, or participant refusal.
2. All low-birth weight children and a 10% sample of non-low-birth weight children were sampled at ages 1-4. Thus growth measures and child IQ measures are available for n=634 cohort members. The analysis uses appropriate sample weighting.
3. The inception cohort includes individuals born from 1962 through 1977, not all of whom were age-eligible for anthropometric measurements at age 2 yr or cognitive testing at ages 3-7 years during the period of field work (1969-1977)

Supplementary Table 3: Adult cognitive achievement by schooling attainment, by site and sex

	n	IQ among those not completing threshold schooling<br>mean; SD	IQ among those completing threshold schooling<br>mean; SD
Brazil females	249	91·8; 12·6	105·6; 13·1
Brazil males	201	93·1; 14·0	109·4; 13·6
Guatemala females	229	92·2; 10·3	105·1; 15·3
Guatemala males	196	96·9, 10·9	109·7; 15·4
Philippines females	590	87·6; 12·0	102·6; 14·5
Philippines males	686	90·4; 11·5	105·3; 13·7
South Africa females	247	88·1; 15·0	102·5; 13·6
South Africa males	216	97·0; 14·2	106·3; 10·8

Threshold schooling: Completion of primary school in Guatemala, secondary school elsewhere·

All differences between those not completing and those completing threshold schooling levels significant at p<0·0001 by unpaired t-test

Data Collection

Those involved in previous rounds of data collection we re-contacted and recruitment and data collection were done by extensively trained field staff under the direction of a highly experienced team leader. A full description of the data collection done in Brazil can be found in Gonçalves, H., Wehrmeister, F. C., Assunção, M. C. F., Tovo-Rodrigues, L., Oliveira, I. O. d., Murray, J., . . . Menezes, A. M. B. (2017). Cohort Profile Update: The 1993 Pelotas (Brazil) Birth Cohort follow-up at 22 years. *International Journal of Epidemiology*, dyx249-dyx249. doi:10.1093/ije/dyx249

In Cebu consenting individuals were interviewed using Computer-Assisted Personal Interviewing (CAPI), followed by Raven's Progressive Matrices administered with pen and paper. The reviewed completed questionnaires/forms were immediately transmitted to the server and Raven's PM responses were encoded at OPS using MS Excel and entries were reviewed by another encoder to ensure correctness. Data processing/cleaning activities (consistency checks, assigning numeric values to text responses, translation, recoding as necessary, etc.) were then

done by the Data Manager in coordination with the Project Coordinator and Investigator. In South Africa data collection was onsite and paperless, and utilised wifi-enabled tablets (Samsung) or laptops (HP) connected to the project database (REDCAP) through respective data collection forms. The REDCAP database is securely housed and backed up on the University of the Witwatersrand server. In Guatemala, information was collected using the RedCap Mobile APP with iPad 5th generation (Model MP2H2LL/A), operating system iOS. Information collected on iPad was then synchronized to INCAP's server every week by on site data manager. Information was reviewed for missing values, outliers or wrongly entered data on a monthly basis. The collected data enters the server exactly as collected. De-identified data was shared with the COHORTS group.
